# Diabetes Mellitus Successfully Treated by Ioduret of Iron

**Published:** 1844-03

**Authors:** 


*Diabetes Mellitus successfully treated by Ioduret of Iron.*—This case was admitted into the Hotel Dieu, having experienced the premonitory symptoms of diabetes three weeks previously, which were now completely confirmed; he passed fifteen quarts of urine daily, which contained a considerable quantity of sugar.

The treatment consisted in restricting him to animal diet, the administration of a bottle of claret daily, with a flask of Bagnol's wine, broth without bread, and lemonade, etc., for drink; he also took four pills, each containing five grains of the ioduret of iron, in the twenty-four hours. Under this mode of treatment the quantity of urine discharged soon diminished; after the third day the man passed only twelve quarts; during the subsequent days the quantity underwent a still greater diminution, and the thirst, together with the other symptoms of diabetes, subsided; the urine discharged exceeded the quantity of fluid ingested by about a pint, and contained but a very small proportion of saccharine matter. On the 30th of September he drank about three quarts of tizan, in addition to his wine and broth, and passed four quarts of urine. From the 30th of September to the 4th of October the treatment was continued, (the number of pills being increased to five) and the improvement of the patient kept pace with it; the quantity of urine passed, at the latter date, was nearly normal, and it contained hardly a trace of sugar: the thirst had ceased, the strength was returning, and the patient was enabled to leave the hospital on the 5th of October in good health. He was seen a few days afterward in town, and continued well.

The ioduret of iron must have had considerable effect in this case, as the usual diet of animal food, etc., recommended in these cases, had previously failed in doing good till combined with this medicine.—*Braith. Ret.*, from *Provin. Med. and Surg. Jour.*, Nov. 5, 1842, p. 112.

